# Machine Learning Model Discriminate Ischemic Heart Disease Using Breathome Analysis

**DOI:** 10.3390/biomedicines12122814

**Published:** 2024-12-11

**Authors:** Basheer Abdullah Marzoog, Peter Chomakhidze, Daria Gognieva, Nina Vladimirovna Gagarina, Artemiy Silantyev, Alexander Suvorov, Ekaterina Fominykha, Malika Mustafina, Ershova Natalya, Aida Gadzhiakhmedova, Philipp Kopylov

**Affiliations:** 1 World-Class Research Center «Digital Biodesign and Personalized Healthcare», I.M. Sechenov First Moscow State Medical University (Sechenov University), 8-2 Trubetskaya Street, 119991 Moscow, Russia; 2 University Clinical Hospital Number 1, Radiology Department, I.M. Sechenov First Moscow State Medical University (Sechenov University), 8-2 Trubetskaya Street, 119991 Moscow, Russia

**Keywords:** breathome, metabolome, PTR-TOF-MS, VOCs, IHD, optimizing, bicycle ergometry

## Abstract

**Background:** Ischemic heart disease (IHD) impacts the quality of life and is the most frequently reported cause of morbidity and mortality globally. **Aims:** To assess the changes in the exhaled volatile organic compounds (VOCs) in patients with vs. without ischemic heart disease (IHD) confirmed by stress computed tomography myocardial perfusion (CTP) imaging. **Objectives:** IHD early diagnosis and management remain underestimated due to the poor diagnostic and therapeutic strategies including the primary prevention methods. **Materials and Methods:** A single center observational study included 80 participants. The participants were aged ≥ 40 years and given an informed written consent to participate in the study and publish any associated figures. Both groups, G1 (*n* = 31) with and G2 (*n* = 49) without post stress-induced myocardial perfusion defect, passed cardiologist consultation, anthropometric measurements, blood pressure and pulse rate measurements, echocardiography, real time breathing at rest into PTR-TOF-MS-1000, cardio-ankle vascular index, bicycle ergometry, and immediately after performing bicycle ergometry repeating the breathing analysis into the PTR-TOF-MS-1000, and after three minutes from the end of the second breath, repeat the breath into the PTR-TOF-MS-1000, then performing CTP. LASSO regression with nested cross-validation was used to find the association between the exhaled VOCs and existence of myocardial perfusion defect. Statistical processing performed with R programming language v4.2 and Python v.3.10 [^R], STATISTICA program v.12, and IBM SPSS v.28. **Results:** The VOCs specificity 77.6% [95% confidence interval (CI); 0.666; 0.889], sensitivity 83.9% [95% CI; 0.692; 0.964], and diagnostic accuracy; area under the curve (AUC) 83.8% [95% CI; 0.73655857; 0.91493173]. Whereas the AUC of the bicycle ergometry 50.7% [95% CI; 0.388; 0.625], specificity 53.1% [95% CI; 0.392; 0.673], and sensitivity 48.4% [95% CI; 0.306; 0.657]. **Conclusions:** The VOCs analysis appear to discriminate individuals with vs. without IHD using machine learning models. **Other**: The exhaled breath analysis reflects the myocardiocytes metabolomic signature and related intercellular homeostasis changes and regulation perturbances. Exhaled breath analysis poses a promise result to improve the diagnostic accuracy of the physical stress tests using machine learning models.

## 1. Introduction

Incredibly, considering exhaled air as the mirror of the health of the organism is a promising approach for the future multifunctional strategy in terms of diagnosis, treatment, prevention, and evaluation of the patients prognosis [1]. Exhaled air contains a plethora of volatile organic compounds (VOCs) that demonstrate the health of the organism, including the health of the cardiovascular system [1,2]. The components of the exhaled air are variable according to the changes in the systems and organs of the body. For instance, changes in the components of exhaled air in a patient with a gastrointestinal tract pathology are different from a patient with a lung pathology. Therefore, we hypothesis that patients with cardiovascular disease have a different chemical biomarkers component in their exhaled air. At the same time, we suggest that patients with ischemic heart disease have different levels of the VOCs in their exhaled air according to the risk of death in the next 10 years using the formula of the European Society of Cardiology (SCORE2, SCORE2-OP, and SMART Risk Score) [3,4,5].

Various types of mass spectrometry have been developed in recent years in hope of accurately analyzing exhaled breath volatile compounds. Mass spectrometry is a unique technique in which atoms and molecules of a sample are ionized, accelerated to MeV energies, and separated according to their momentum, charge, and energy, allowing high discrimination for the measurement of isotope abundances [6].

Exhaled air analysis has been performed in patients with different pathologies including chronic obstructive lung disease, cancer, asthma, lung cancer, diabetes, arthritis, heart failure, gastric cancer, chronic kidney disease, colorectal cancer, hepatocellular carcinoma, malignant pleural mesothelioma, bladder cancer, pancreatic ductal adenocarcinoma, gastro-oesophageal cancer, peritonitis-shock, head and neck squamous cell carcinoma, multiple sclerosis, and Parkinson’s disease [7,8,9,10,11,12,13,14,15,16,17,18,19,20,21,22,23,24,25,26,27,28,29,30,31,32,33,34,35,36,37,38,39,40,41,42,43,44,45].

Despite the current advances in technologies and therapeutic strategies, identifying the origin of the molecules in the exhaled air analysis remains a challenge for scientists. The compounds of the exhaled air depend on several factors, both exogenous and endogenous. Part of the endogenous factors is the presence of pathologies in the organism, including ischemic heart disease. However, exogenous factors play a critical role in the components of the exhaled air such as smoking. Where smoking is associated with 80 molecules (unsaturated hydrocarbons; 29 dienes, 27 alkenes, and 3 alkynes) in the analysis of exhaled breath compared to non-smokers [46].

Additionally, the precision and greater chance of detection of some VOCs require special preconditions including the selection of the relevant breath fraction, the type of breath collection container (if used), and the preconcentration technique [47]. Sampling of the late expiratory breath is preferred to obtain a greater endogenous contribution [47]. Additionally, the breath collection containers must not have a condensation effect on the collected sample. For these reasons, the scientific community requires further development a protocol in the preferred methods for collecting, processing, evaluating the results of the exhaled breath air analysis [47].

Cardiovascular disease (CVD) is the leading cause of mortality and morbidity in our era despite the current advances in therapeutic strategies and technologies [48]. Unfortunately, each second a person dies due to CVD globally [49]. Moreover, ischemic heart disease ranks first on the list of most frequent causes of mortality and morbidity among cardiovascular diseases. In patients with ischemic heart disease, including energy deprivation, the changes in the exhaled breath analysis are reflected in various ways, particularly through the detection of volatile organic compounds (VOCs) and other biomarkers. However, myocardiocytes activate specific signaling pathways to survive and prolong the resistant period through elevating the necrosis threshold and transforming the myocardiocyte into dormant status. Furthermore, myocardiocytes upregulate functionality of autophagy function to improve the cellular antioxidant defense system and reduce energy expenditure [50,51,52,53].

Current research on using exhaled breath analysis for diagnosis, follow-up of treatment regime, early prevention, and prognosis determination of prognosis in ischemic heart disease patients remains in the womb of development.

The study sought to improve the diagnosis of ischemic heart disease during physical exertion test using PTR-TOF-1000 real-time mass spectrometry (MS).

## 2. Materials and Methods

### 2.1. Study Design

A prospective, non-randomized, minimally invasive, single-center, case–control cohort study included patients (male and female) aged ≥ 40 years because the risk of coronary heart disease increases dramatically over the age of forty. The recruitment of participants took place from 27 October 2023 to 28 October 2024 at the University Clinical Hospital No1 of Sechenov University. Initial data of patients with pathology are obtained from the Department of Cardiology, and for healthy patients by invitation.

The study was conducted in accordance with the standards of Good Clinical Practice and the principles of the Declaration of Helsinki. The study protocol was approved by the local ethics committee (protocol No 19-23 dated 26 October 2023). The study is registered on the clinicaltrials.gov website (NCT06181799). Following the completion of the data collection phase, a database was established, and statistical analyses were carried out. The design of the study and methods of statistical data processing corresponded to the goals and objectives of the study.

Before being included in the study, patients gave written informed consent to participate in the study, consent to the processing of personal data indicated by the doctor conducting the study.

The results of the stress test (bicycle ergometry) and myocardial perfusion are interpreted by doctors with at least 5 years of experience in each field, a radiologist, a functional diagnostician, respectively.

The first group comprised 31 participants who exhibited stress-induced myocardial perfusion defect on the stressed computer tomography myocardial perfusion (CTP) imaging. This was achieved using contrast-enhanced multi-slice spiral computed tomography (CE-MSCT) and adenosine triphosphate (ATP) as a stress test.

**Figure 1:**
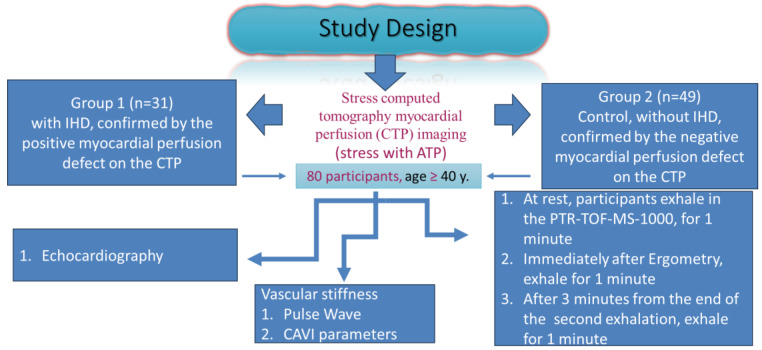
Diagrammatic presentation of the study. The patients exhale in mass spectrometer (PTR TOF-1000 (IONICON PTR-TOF-MS—Trace VOC Analyzer). Subsequently, participants pass exercise bicycle ergometry (on SCHILLER device; Bruce protocol or modified Bruce protocol). Then, breathe immediately after the end of the test (second breath), and after three minutes from the end of the second breath, the participants again exhale in the same mass spectrometer using single time tube.

In contrast, the second group included 49 participants who did not exhibit stress-induced myocardial perfusion defect on the stressed CTP imaging. This was also achieved using CE-MSCT and ATP as a stress test. Additionally, the health of the participants was confirmed by the medical history, previous medical analyses, and retrospective consultation. The study included both males and females, and the age of the participants ≥ 40 years. All the participants assessed their anthropometric measurements, blood pressure and pulse rate before starting the study, at rest (Figure 1).

The sample size was reached after calculation of the related mean sample power analysis and Pearson correlation power analysis using SPSS program (Table 1A,B).

### 2.2. Data Collection

The study evaluated continuous and categorical variables. The continuous variables included age, pulse at rest, systolic blood pressure (SBP) at rest, diastolic blood pressure (DBP) at rest, body weight, height, maximum heart rate (HR) on physical stress test, watt (WT) on physical stress test, metabolic equivalent (METs) on physical stress test, reached percent on physical stress test, ejection fraction (EF %) on echocardiography, estimated vessel age, right cardio-ankle vascular index (R-CAVI), left Cardio-ankle vascular index (L-CAVI), mean CAVI (=(right-CAVI + left-CAVI)/2), right ankle-brachial index (RABI), left ankle-brachial index (LABI), mean ankle-brachial index (ABI), mean SBP brachial (SBPB) (=(right SBPB + left SBPB)/2), mean DBPB (=(right DBPB + left DBPB)/2), BP right brachial (BPRB) (=(SBP + DBP)/2), BP left brachial (BPLB) (=(SBP + DBP)/2), mean BPB (=(BPRB + BPLB)/2), BP right ankle (BPRA) (=(SBP + DBP)/2), BP left ankle (BPLA) (=(SBP + DBP)/2), mean BPA (=(BPRA + BPLA)/2), right brachial pulse (RTb), left brachial pulse (LTb), mean Tb (=(LTb + RTb)/2), right brachial-ankle pulse (Tba), left brachial-ankle pulse (Tba), mean Tba (=(left Tba + right Tba)/2), length heart-ankle (Lha in cm), heart-ankle pulse wave velocity (haPWV = Lha/(mean left Tba + mean right Tba); m/s), β-stiffness index from PWV (=2*1050*(haPWV)^2*LN((mean SBPB*133.32)/(mean DBPB*133.32))/((mean SBPB*133.32) − (mean DBPB*133.32))), creatinine (µmol/L), and eGFR (2021 CKD-EPI Creatinine). Categorical variables included gender, obesity stage, smoking, concomitant disease, coronary artery, hemodynamically significant (>60%), myocardial perfusion defect after stress ATP, myocardial perfusion defect before stress ATP, atherosclerosis in other arteries (Yes/No), carotid atherosclerosis, brachiocephalic atherosclerosis, arterial hypertension (AH), stage of the AH, degree of the AH, risk of cardiovascular disease (CVD), stable coronary artery disease (SCAD), functional class (FC) by Watt and by METs, reaction type to stress test (positive/negative), reason of discontinuation of the stress test, CAVI degree, and ABI degree.

The selection criteria of the participants are represented in the table below (Table 2).

The current paper is a PhD work by MD. Basheer A. Marzoog. This study was registered at the clinicaltrails.gov (NCT06181799), and was approved by the Sechenov University, Russia, from “Ethics Committee Requirement № 19-23 from 26 October 2023”. Written consent was obtained from the study participants for publication of any of the obtained results including figures.

### 2.3. Instrumental Methods

#### 2.3.1. Mass Spectrometry

All participants, at rest, passed real-time mass spectrometry (MS) within one minute using a PTR TOF-MS-1000 (IONICON PTR-TOF-MS-1000 Trace VOC Analyzer, Eduard-Bodem-Gasse 3, 6020 Innsbruck, Austria (Europe). The analysis of exhaled air was carried out in the hospital in the morning, on an empty stomach, without toothbrushing. All participants abstained from food and liquids (except water) and exercise training for 6–8 h before breathing [54]. Participants used disposable and sterile mouthpieces, and according to the manufacturer’s instructions, additional filters were not required. All participants breathed into the PTR-TOF-MS-1000 for 1 min (during this time from 12 to 16 exhalation cycles are analyzed). The ionized molecules were separated by their *m*/*z* and subsequently detected. Full scan mass spectra were obtained in the 10–685 mass-to-charge ratio (*m*/*z*) with a scan time of 1000 ms and primary ion H_3_O^+^. The temperature of T-Drift and T-Inlet was 80°.

#### 2.3.2. Vessel Stiffness Measurement

Both groups passed a vessel stiffness test and pulse wave recording as well as vascular age by using Fukuda Denshi device (VaSera VS-1500; Tokyo, Japan). Cuffs were placed to assess the vascular stiffness and the vascular age as well as the ancle-brachial index.

Cuffs fit the size of the arms and ankles of the patients. Electrodes attach to the two arms, and a microphone for cardio-phonogram measurements fix with double-sided tape over the sternum in the second intercostal space. Cardio-ankle vascular index (CAVI parameter) reflects the overall stiffness of the aorta, femoral artery and tibial artery, and is theoretically not affected by blood pressure [55]. CAVI measurements considered valid only when obtained during at least three consecutive heartbeats [55]. These CAVI measurements exclude vascular pathology and determine the biological age of the blood vessels. The measurement of the vascular stiffness and estimated vascular age is to determine the state of the non-coronary arteries.

#### 2.3.3. Physical Exertion Test

After the first exhalation into the real time mass spectrometry, PTR-TOF-MS-1000, at rest in a room with considerable ideal in terms of the pollution in the atmosphere, hospital environment, participants passed exercise bicycle ergometry (on SCHILLER CS200 device; Bruce protocol or modified Bruce protocol) test to evaluate the response to physical activity. And just after completing the exercise test, the participants exhaled a second time into the same real-time mass spectrometry, within one minute. And a third time exhaled into the same mass spectrometry after three minutes from the end of the second breathing, within one minute. According to the results of the metabolic equivalent, Mets-BT (BT), the angina functional class (FC) in participants with positive stress test results was determined as follows: ВТ/Mets < 50/<4 FC-III, ВТ/Mets 50–100/4–7 FC-II, ВТ/Mets > 100/7 FC-I. During the bicycle ergometry test, the participants monitored with 12-lead ECG and manual blood pressure measurement, once every 2 min, close to the end of each stage.

The ergometry procedure was discontinued if there was an increase in systolic blood pressure ≥ 220 mmHg or horizontal or downsloping ST segment depression on the ECG ≥ 1 mm, typical heart pain during test, ventricular tachycardia or atrial fibrillation, or other significant heart rhythm disorders were found. Moreover, the procedure was stopped if the target heart rate (≥86% of the 220-age) was reached.

#### 2.3.4. Stressed Computer Tomography Myocardial Perfusion (CTP) Imaging

Before performing the stressed computer tomography with myocardial perfusion imaging, all the participants presented results of the venous creatinine level, eGFR (estimated glomerular filtration rate) according to the 2021 CKD-EPI creatinine > 30 mL/min/1.73 m^2^, according to the recommendation for using this formula by the National Kidney Foundation and the American Society of Nephrology [56,57,58,59].

The participants of both groups obtained catheterization in the basilar vein or the radial vein for injection of contrast and Natrii adenosine triphosphate (10 mg/1 mL) to induce pharmacological stress test to the heart by increasing heart rate. Then, the catheter was used for the contrast injection during the procedure of the computer tomography.

To prepare the Natrii Adenosine Triphosphate, 3 mL of Adenosine Triphosphate was diluted in 17 mL of isotonic Sodium Chloride solution, 0.9%. The injected volume of the diluted drug in milliliters is calculated by body weight. For 1 dose, take 3 mL of adenosine triphosphate (3 ampoules of each 1 mL (10 mg)) and 17 mL of isotonic solution of sodium chloride, 0.9%, in one syringe, 20 mL. For one patient, manually inject intravenously (IV) through the already inserted catheter at a rate of 300 μg/kg/2 min, depending on weight: 60 kg = 12 mL, 70 kg = 14 mL, 80 kg = 16 mL, and 100 kg = 20 mL of the full dose.

Stress computed tomography myocardial perfusion (CTP) imaging (performed on Canon device with 640 slice, 0.5 mm thickness) with contrast (Omnipaque, 50 mL). Firstly, make an image to evaluate the calcification level in the valves and the ascending aorta. Then, inject the contrast and make a resting image for myocardial perfusion, then the patient continues lying on the apparatus for 20 min and then inject the Natrii Adenosine Triphosphate (10 mg/1 mL) into the catheter, during 2 min, according to body weight to cause a pharmacological stress test to the heart. Then, make an image of the myocardial perfusion after stress test immediately; the image must be taken in less than 30 s.

### 2.4. Statistical Analysis

For quantitative parameters, the nature of the distribution (using the Shapiro–Wilk test), the mean, the standard deviation, the median, the interquartile, the minimum, and maximum values were determined. For categorical and qualitative features, the proportion and absolute number of values were determined.

Comparative analysis for normally distributed quantitative traits was carried out on the basis of Welch’s *t*-test (2 groups); for abnormally distributed quantitative traits, use the Mann–Whitney U-test (2 groups).

Comparative analysis of categorical and qualitative features was carried out using the Pearson X-square criterion; in case of its inapplicability, use the exact Fisher test.

For exhaled air values, baseline values (prefixed with “l0_”) were used, and deltas between and immediately after exertion (l1) and after 2nd exhalation, as well as between and after 2nd exhalation and immediately after exertion, were calculated:

l0=before

l1=after 1

l3=after 3

Calculation of delts:

$$dltlos_{01}=l1-l0$$

$$dltlos_{03}=l3-l0$$

$$dltlos_{13}=l3-l1$$

Statistical processing carried out using the R programming language v4.2, Python v.3.10 [^R], Statistica 12 program. (StatSoft, Inc. Tulsa, OK, USA, (2014). STATISTICA (data analysis software system), version 12. www.statsoft.com), and IBM SPSS v.28. *p* considered statistically significant at <0.05.

### 2.5. Outcome and Feature Selection with Cross-Validation Using Machine Learning Models

According to the number of observations (*n* = 80), random sampling of 2/3 of the available sample for predictor selection was performed for 1000 repetitions to evaluate the performance of the predictors. Data preprocessing at each iteration involved normalization and iterative imputation using Bayesian ridge regression for quantitative data. There were no categorical or binary features. At each iteration, a classifier was built using the gradient boosting algorithm, which made it possible to calculate feature importances 1000 times. Then, feature importances medians were calculated for each factor, and predictors were ranked from the highest median values to the lowest.

Ten selected predictors were included in a new pipeline, the same data preprocessing was performed, then a classifier was built using the gradient boosting algorithm. Leave-one-out cross-validation was used. After that, the area under the curve, AUC, was calculated, and the optimal threshold was selected for calculating sensitivity and specificity, positive and negative prognostic values. The obtained area under the curve was compared with the result of stress test using the McNemar criterion. This procedure was performed separately for the obtained exhaled breath data without the other clinical data.

## 3. Results

### 3.1. The Cohort

The primary included number in the study is 101 individuals, excluding 21 (either discontinued the study by their decision or excluded due to the detection an exclusion criteria).

The prospective study involved 80 participants. According to the results of the CTP, the participants are divided into two groups. The first group of participants were those with stress-induced myocardial perfusion defect (*n* = 31) and the second group were those without stress-induced myocardial perfusion defect (*n* = 49) on the CTP.

### 3.2. Descriptive Statistics Results

The descriptive characteristics of the sample were shown as both groups and then each group separately in tables for a full representation of the results. The characteristics of the continuous variables of the sample described in the below tables (Table 3A,B).

The comparative characteristics of the sample represented in the below tables based on the presence or absence of the stress-induced myocardial perfusion defect of the CTP imaging with the adenosine triphosphate (Table 4A,B).

A Supplementary File was attached to demonstrate all the statistically significant differences between continuous variables using the binary categorical variables as a classifier (Supplementary S1).

### 3.3. The Diagnostic Accuracy of the Bicycle Ergometry

We examined the diagnostic accuracy of a standard exercise test on a bicycle ergometer. In the ROC analysis, where the predictor was the result of a sample with the results of the physical exertion “Reaction_type” = ‘Positive’, and the target variable was Myocardial_perfusion_defect_after_stress_ATP, the following results were obtained (Table 5).

### 3.4. Exhaled Breath Biomarkers Diagnostic Accuracy

LASSO logistic regression and the XGBoost algorithm were employed to identify the most influential volatile organic compounds (VOCs) associated with IHD. Prior to analysis, the VOC values were normalized. A five-fold cross-validation approach was utilized for both classifier training and feature selection. Considering the limited sample size, no additional validation or splitting techniques were performed.

In LASSO regression, feature selection was based on the absolute value of regression coefficients. Conversely, XGBoost employed the magnitude of the gain score as a criterion [60]. The objective was to select features such that including the N-strongest predictors in the classifier resulted in an AUC of at least 0.98. Subsequently, the quality of the fitted classifiers was assessed using the area under the curve (AUC), sensitivity, and specificity. The following predictors were selected for VOCs, the top 10 based on the median feature importances are presented below (Table 6).

The model was then rebuilt as follows: the top five predictors from Table 6 had the highest mathematical importance according to the built model taken and included in the new LASSO regression model.

Then, the leave-one-out cross-validation procedure was performed, which allowed us to obtain approximate estimates of sensitivity, specificity, and positive and negative prognostic value. At each iteration of leave-one-out cross-validation, the quantitative predictors were normalized. The quality of the classification is shown in the table below (Table 7).

**Figure 2:**
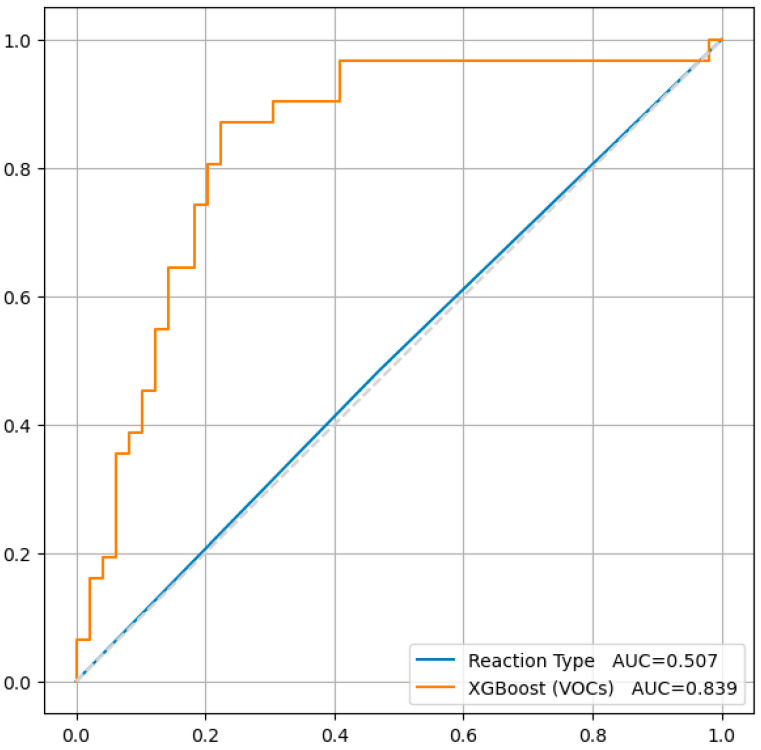
There is statistically significant difference between the results of the diagnostic accuracy of the load test (50.7%) and the built model (83.9%), based on our study results. Obviously, the model has better predictive properties. *p* value = 0.003.

Confidence results are calculated using a bootstrap. Comparison with load results was carried out using the McNemar test [61] (Figure 2).

### 3.5. The Found m/z Annotation

The presented *m*/*z* (mass/charge) ratio has been translated into chemical formula (if existent in the literature or library of the IONICON PTR-TOF-MS-1000 device). Data preprocessing and the annotation of molecular formulae are presented in the below table (Table 8).

An attempt is made to estimate the correlations of delts with each other. The analysis was performed between each pair of delts according to Spearman correlation test. In our study, there are a small number of patients (*n* = 80), but a significant number of predictor variables. Routine Spearman correlation analysis revealed a huge number of correlations, while it is impossible to understand how predictors behave together. To assess the endpoint’s relationship with predictors, we used LASSO to identify the main predictors and reduce multicollinearity.

## 4. Discussion

In light of the presented results, the VOC concentration differences are statically different between the two groups (Group 1 with positive CPT/Group 2 with negative CPT). Especially, when comparing the concentration of the VOCs before and after performing the physical exertion test (bicycle ergometry), presented as a delts. Suggesting that changes in the concentration of the VOCs were associated with the agitation of the ischemic heart disease and not for other reasons. Moreover, atherosclerosis of the other arteries, brachiocephalic or carotid, has no statistically significant difference between the groups, which confirms that the change in the concentrations of the VOCs was due to the worsening of the ischemic heart disease in terms of perturbance of the myocardial nourishment.

The changes in the concentration of the stated chemical substances statistically and significantly changed between the third and the second/first breath which indicates that these substances associated with the worsening of myocardial nourishment.

Fluctuation in the concentrations of VOCs is of clinical importance in the context of improvement of the diagnostic accuracy of ischemic heart disease in the clinical settings. This clinical importance is presented by the dramatic enhancement of the diagnosis of IHD in combination of physical exertion test (bicycle ergometry) and exhaled breath analysis.

The presented chemical substance is represented as a ratio to charge. Accordingly, if the mass/charge is known, we present it as a name of the chemical substance. In case the mass/charge is unknown, we leave the ratio and write the chemical formula of this chemical substance.

The pathomorphological changes in ischemic heart disease represented by the metabolic acidosis of the ischemic myocardiocytes and further elaboration of the pathological cardiac metabolic changes in coronary circulation. The ischemic myocardiocytes suffer from pathophysiological changes in terms of the intercellular metabolism, which is interrupted by the disturbance in the regulation mechanisms of the intracellular homeostasis. These physiopathological changes represented by elevation of the biomarkers of the oxidative stress and the ischemia–reperfusion injury.

To improve cardiometabolism, exogenous application of activators (glycolysis), activation of Sirt1 or 3 (activation of autophagy; by NAD^+^ administration: deacetylation), ketone oxidation, activation of the pyruvate dehydrogenase complex (glucose oxidation), and activation of the hexosamine biosynthesis pathway (O-GlcNAcylation; administration of glucosamine/glucose) pose cardio-therapeutic effects [63]. On the contrary, inhibition of mitochondrial oxygen consumption, malate–aspartate shuttle, mitochondrial succinate metabolism (malonate), fatty acid oxidation (CD36 inhibitors, malonyl-CoA decarboxylase inhibitors), and inhibiting destabilization of FOF1-ATPase dimers or maintaining the association of hexokinase II or creatine kinase with mitochondria to protect the cristae structure of the mitochondria [63].

The potential source of these VOCs in the exhaled breath analysis of patients with ischemic heart disease can be assessed based on the pathomorphological changes in the ischemic heart tissue. The found VOCs include C_4_H_8_O^+^, 2-Pentanone or 3-methyl-2-butanone, *m*/*z* 87.9337, C_2_H_7_NO_3_H^+^, and *m*/*z* 144.9178.

The study lacks the external validation due to the technical limitations represented by the fact that the PTR-TOF-MS-1000 device is the only one in Russia and no other works globally performed using this device on patients with IHD confirmed by the myocardial perfusion defect on the CTP imaging.

The study compared the exhaled VOCs analysis with the physical exertion (bicycle ergometry) test due to the fact that these tests are the most widely acceptable test for the diagnosis of the IHD in the clinical practice, as a non-invasive diagnostic method. Whereas the other methods are considered to be additional and not the gold standard for IHD diagnosis, excluding the coronary angiography with fractional flow reserve and the CTP imaging with pharmacological stress test [64]. Moreover, the other tests are not available at all the medical facilities and usually are expensive and require relatively long waiting times and preparation regarding the CTP imaging of the myocardium and the coronary angiography.

Therefore, we suggest using exhaled breath analysis as an alternative for risk stratification and diagnosis of IHD in a rapid and cost-effective manner. Moreover, it is advisable to develop a more selective and specific electronic nose (e-nose) that is specific for the found VOCs in IHD patients. This returns to the fact that PTR-TOF-MS-1000 is a very expensive device and not available at all the medical facilities to make it possible to use for routine screening for IHD [65,66].

Regarding the potential origin of the obtained VOCs, three hypotheses are suggested [2,65,66,67]. The first hypothesis is the gut microflora dysbiosis in patients with IHD released into their metabolites either directly through the esophagus then with the exhaled breath or into the blood circulation then bypassed by some metabolic changes in the blood circulation and then evacuated with the exhaled breath through the lung.

The second hypothesis is the changes in the blood vessels in terms of endothelial dysfunction and atherosclerosis formation. These changes include the release of the metabolites from dysfunctional endothelial cells, the atherosclerosis forming cells and or normal microflora in the atherosclerosis plaque into the blood circulation then evacuated with the exhaled breath [68].

The third hypothesis, is the that these VOCs originated from the ischemic myocardiocytes, as a metabolic byproduct of the ischemic myocardiocyte metabolic changes [51,52,65,66,69,70,71,72,73,74].

Individual variability due to physiological differences, diet, exercise, environmental exposures, and health conditions can significantly impact the concentrations and profiles of exhaled VOCs. Understanding these factors is essential for improving the accuracy and reliability of breath analysis as a diagnostic tool for various diseases [65,66].

While the study indicates promising accuracy for the machine learning model in detecting IHD through the exhaled breath analysis, the reproducibility of these results over time remains a concern. Further research is needed to assess how consistent VOC profiles are across multiple tests and to identify strategies to minimize variability. This could involve standardizing testing conditions, controlling for dietary and environmental factors, and establishing protocols for repeated measurements using the e-nose [65,66].

## 5. Conclusions

Exhaled breath analysis can improve the diagnostic accuracy of the bicycle ergometry in the diagnosis of IHD. Patients with discrepancy between supply and demand of heart muscle tissue with blood experience elevation in VOCs include C_4_H_8_O^+^, 2-Pentanone or 3-methyl-2-butanone, *m*/*z* 87.9337, C_2_H_7_NO_3_H^+^, and *m*/*z* 144.9178. Further investigation is required to reveal the full pilot of the exhaled breath analysis VOCs in patients with IHD.

## Figures and Tables

**Table 1:** (**A**)—The related mean sample power analysis and (**B**)—Pearson correlation power analysis.

**(A) Power Analysis Table**						
	N ^b^	Actual Power ^c^	Test Assumptions			
			Power		Effect Size	Sig.
Test for Mean Difference ^a^	54	0.950	0.95		0.5	0.05
a. Two-sided test.						
b. Number of pairs.						
c. Based on noncentral t-distribution.						
**(B) Power Analysis Table**						
	N	Actual Power ^b^	Test Assumptions			
			Power	Null	Alternative	Sig.
Pearson Correlation ^a^	46	0.954	0.95	0	0.5	0.05
a. Two-sided test.						
b. Based on Fisher’s z-transformation and normal approximation with bias adjustment.						

**Table 2:** The selection criteria of the study participants.

Inclusion Criteria	Exclusion Criteria	Non-Inclusion Criteria
Participants age ≥ 40 years.	Poor single-channel ECG and pulse wave recording quality	Pregnancy and breast feeding.
Participants with intact mental and physical activity.	Failure of the stress test for reasons unrelated to heart disease	Diabetes mellitus
Written consent to participate in the study, take blood samples, and anonymously publish the results of the study.	Reluctance to continue participating in the study.	Presence of signs of acute coronary syndrome (myocardial infarction in the last two days), history of myocardial infarction;
The participants of the experimental group are individuals with coronary artery disease, confirmed by myocardial perfusion defect on the adenosine triphosphate stress myocardial perfusion computed tomography, and confirmed by medical history, previous medical tests, and retrospective interview of participants.		Active infectious and non-infectious inflammatory diseases in the exacerbation phase;
		Respiratory diseases (bronchial asthma, chronic bronchitis, cystic fibrosis);
		Acute thromboembolism of pulmonary artery branches;
		Aortic dissection;
		Critical heart defects;
		Active oncopathology;
		Decompensation phase of acute heart failure and III-IV stage of heart failure;
		Neurological pathology (Parkinson’s disease, multiple sclerosis, acute psychosis, Guillain-Barré syndrome);
		Cardiac arrhythmias that do not allow exercise ECG testing (Wolff-Parkinson-White syndrome, Sick sinus syndrome, AV block of II-III-degree, persistent ventricular tachycardia);
		Diseases of the musculoskeletal system that prevent passing a stress test (bicycle ergometry);
		Allergic reaction to iodine and/or adenosine triphosphate.

**Table 3:** (**A**): the features (descriptive statistics) of the continues variables of the whole sample represented in the table. (**B**): the features (descriptive statistics) of the categorical variables of the whole sample represented in the table. Abbreviations: METs: metabolic equivalent; CPT: stress myocardial perfusion computer tomography imaging.

(**A**)				
Variable	Mean	Minimum	Maximum	Std. Dev.
Age	56.28	40.24	77.94	10.601
Pulse rate at rest	70.29	49.00	93.00	9.559
SBP at rest	123.16	54.00	159.00	15.437
DBP at rest	80.61	60.00	122.00	11.238
Body weight	77.92	52.50	140.00	16.236
Height	169.95	148.00	190.00	8.835
BMI	26.93	18.49	48.44	4.901
Pulse rest (Cardio-Qvark^®^)	69.68	49.00	99.00	9.260
Pulse after stress (Cardio-Qvark^®^)	86.89	63.00	115.00	10.579
Goal heart rate	163.72	142.06	179.76	10.601
Max HR	146.25	108.00	199.00	14.111
Reached %	89.53	64.89	135.25	9.166
WT	125.63	75.00	250.00	44.111
METs	6.67	2.90	11.90	1.977
EF (%)	64.52	55.00	73.00	4.388
eVessel age	56.50	20.00	80.00	13.520
R-CAVI	8.21	4.80	15.10	1.379
L-CAVI	8.18	4.80	14.90	1.299
Mean CAVI	8194	4800	15,000	1331
RABI	1.15	0.86	1.40	0.088
LABI	1.15	0.89	1.42	0.084
Mean SBP B	134.38	105.50	169.00	13.086
Mean DBP B	85.28	60.50	104.50	8.358
BP RB (=(SBP + DBP)/2)	103.98	75.00	137.00	11.547
BP LB (=(SBP + DBP)/2)	104.54	71.00	136.00	10.534
Mean BP B	104.26	73.00	136.50	10.772
BP RA (=(SBP + DBP)/2)	108.39	80.00	137.00	12.553
BP LA (=(SBP + DBP)/2)	108.63	81.00	138.00	11.519
Mean BP A	108.51	81.50	135.00	11.468
Mean ABI	1.15	0.88	1.41	0.081
RTb	80.53	59.00	152.00	13.441
LTb	77.26	58.00	128.00	14.012
Mean Tb	78.89	59.00	132.50	12.547
Right Tba	86.26	23.00	117.00	16.232
Left Tba	86.40	24.00	114.00	14.685
Mean Tba	86.33	23.50	115.00	15.358
Lha (cm)	148.17	130.33	164.47	7.182
haPWV (m/s)	0.91	0.67	1.42	0.124
β-stiffness index from PWV	2.83	1.21	7.04	0.813
Creatinine (µmol/L)	82.74	53.90	138.00	16.014
eGFR (2021 CKD-EPI Creatinine)	85.31	45.40	113.70	14.684
(**B**)				
Index	Factor		Absolute value (relative value %)	
Gender	F		39 (48.75%)	
	M		41 (51.25%)	
Obesity stage	Normal		30 (37.500%)	
	Overweight		29 (36.25%)	
	1 degree		20 (25.00%)	
	3 degree		1 (1.25%)	
Smoking	Yes		14 (17.50%)	
	No		66 (82.50%)	
Concomitant diseases	Yes		41 (51.25%)	
	No		35 (43.75%)	
	Missing data		4 (5.00%)	
Atherosclerosis of the coronary artery	Yes		31 (38.75%)	
	No		49 (61.25%)	
Hemodynamically significant coronary artery atherosclerosis on the CTP (>60% stenosis)	Yes		9 (11.25%)	
	No		71 (88.75%)	
Stress-induced myocardial perfusion defect on the CTP	Yes		31 (38.75%)	
	No		49 (61.25%)	
Myocardial perfusion defect before Stress ATP on the CTP	Yes		26 (32.50%)	
	No		54 (67.50%)	
Atherosclerosis in other arteries	Yes		41 (51.25%)	
	No		32 (40.00%)	
	Missing data		7 (8.75%)	
Atherosclerotic vascular (Namely)	Carotid		1 (1.25%)	
	Carotid and Brachiocephalic bifurcation		41 (51.25%)	
	Missing data		38 (47.50%)	
Carotid artery atherosclerosis	Yes		39 (48.75%)	
	No		34 (42.50%)	
	Missing data		7 (8.750%)	
Brachiocephalic artery atherosclerosis	Yes		37 (46.25%)	
	No		36 (45.00%)	
	Missing data		7 (8.75%)	
Arterial Hypertension	Yes		40 (50.00%)	
	No		40 (50.00%)	
Stage of the arterial hypertension	I		5 (6.25%)	
	II		20 (25.00%)	
	III		16 (20.00%)	
Degree of hypertension	Degree 1		19 (23.75%)	
	Degree 2		13 (16.25%)	
	Degree 3		9 (11.25%)	
Risk of cardiovascular disease	Low		27 (33.75%)	
	Moderate		27 (33.75%)	
	High		18 (22.50%)	
	Very high		8 (10.00%)	
SCAD from anamnesis	Yes		3 (3.75%)	
	No		29 (36.25%)	
	Missing data		48 (60.00%)	
Blood pressure reaction type on stress test	Asthenic		4 (5.00%)	
	Hypotonic		4 (5.00%)	
	Hypertonic		8 (10.00%)	
	Normotonic		64 (80.00%)	
Functional class by Watt	FC-I		8 (10.00%)	
	FC-II		9 (11.25%)	
	No SCAD		63 (78.75%)	
Functional class by METs	FC-I		6 (7.50%)	
	FC-II		10 (12.50%)	
	FC-III		1 (1.25%)	
	No SCAD		63 (78.75%)	
Reaction type to stress test (positive/negative)	Negative		42 (52.50%)	
	Suspected		21 (22.5%)	
	Positive		17 (21.25%)	
Reason of discontinuation of the stress test	Horizontal ST depression > 1 mm		8 (10%)	
	Reach goal HR		72 (90%)	
Tolerance to exertion on stress test	Low		2 (2.50%)	
	Moderate		43 (53.75%)	
	Close to high		8 (10.00%)	
	High		16 (20.00%)	
	Very high		11 (13.75%)	
CAVI degree	Normal (<8)		36 (45.00%)	
	Borderline (8–9)		22 (27.50%)	
	Pathological (>9)		22 (27.50%)	
ABI degree	Normal		76 (95.00%)	
	Borderline		2 (2.50%)	
	Abnormal		1 (1.25%)	
	Noncompressible		1 (1.25%)	
Biological estimated vascular age	Normal		45 (56.25%)	
	High		35 (43.75%)	
CKD stage	I		35 (43.75%)	
	II		41 (51.25%)	
	IIIa		4 (5.00%)	

**Table 4:** (**A**): categorical variables presented in absolute and relative values of the study for true incidence of the stated factor. X^2^ test used as a comparative test. * Values statically significant difference. Abbreviations: METs: metabolic equivalent; CPT: stress myocardial perfusion computer tomography imaging. (**B**): The continuous variables of the sample presented as a mean ± standard deviation (Std. dev.), Student test as independent variables used. * Values statically significant difference. Abbreviations: SBP: systolic blood pressure; DBP: diastolic blood pressure; BMI: body mass index; HR: heart rate; METs: metabolic equivalent; R-CAVI: right Cardio-ankle vascular index; L-CAVI: left Cardio-ankle vascular index; RABI: right ankle-brachial index; LABI: left ankle-brachial index; SBP B: systolic blood pressure brachial; DBP B: diastolic blood pressure brachial; BP RB: blood pressure right brachial; BP RA: blood pressure right ankle; BP LA: blood pressure left ankle; BP A: blood pressure ankle; ABI: ankle-brachial index; RTb: right brachial pulse; LTb: left brachial pulse; Tb: mean brachial pulse; Tba: mean brachial-ankle pulse; Lha (cm): length heart-ankle; haPWV (m/s): heart-ankle pulse wave velocity.

(**A**)				
Index	Factor	Group 1 (*n* = 31). Positive CTP	Group 2 (*n* = 49). Negative CTP	*p*
Gender	F	17 (54.83%)	22 (44.89%)	0.387
	M	14 (45.16%)	27 (55.10%)	
Obesity stage	Normal	12 (38.70%)	18 (36.73%)	0.988
	Overweight	11 (35.48%)	18 (36.73%)	
	1 degree	8 (25.80%)	12 (24.48%)	
	3 degrees	0 (0.0%)	1 (2.04%)	
Smoking	Yes	7 (22.58%)	7 (14.28%)	0.342
	No	24 (77.41%)	42 (85.71%)	
Concomitant diseases	Yes	15 (48.38%)	26 (53.06%)	0.835
	No	12 (38.70%)	23 (46.93%)	
	Missing data	4 (12.90%)	0 (0.00%)	
Atherosclerosis of the coronary artery	Yes	16 (51.61%)	15 (30.61%)	0.061
	No	15 (48.38%)	34 (69.38%)	
Hemodynamically significant coronary artery atherosclerosis on the CTP (>60% stenosis)	Yes	8 (25.80%)	1 (2.04%)	0.002 *
	No	23 (74.19%)	48 (97.95%)	
Stress-induced myocardial perfusion defect on the CTP	Yes	31 (100%)	0 (0.0%)	<0.001 *
	No	0 (0.0%)	49 (100%)	
Myocardial perfusion defect before Stress ATP on the CTP	Yes	21 (67.74%)	5 (10.20%)	<0.001 *
	No	10 (32.25%)	44 (89.79%)	
Atherosclerosis in other arteries	Yes	22 (70.96%)	19 (38.77%)	0.006 *
	No	7 (22.58%)	25 (51.02%)	
	Missing data	2 (6.45%)	5 (10.20%)	
Atherosclerotic vascular (Namely)	Carotid	1 (3.22%)	0 (0.00%)	0.347
	Carotid. Brachiocephalic bifurcation	21 (67.74%)	19 (38.77%)	
	Missing data	9 (29.03%)	30 (61.22%)	
Carotid artery atherosclerosis	Yes	20 (64.51%)	19 (38.77%)	0.015 *
	No	8 (25.80%)	26 (53.06%)	
	Missing data	3 (9.67%)	4 (8.16%)	
Brachiocephalic artery atherosclerosis	Yes	18 (58.06%)	19 (38.77%)	0.067
	No	10 (32.25%)	26 (53.06%)	
	Missing data	3 (9.67%)	4 (8.16%)	
Arterial Hypertension	Yes	19 (61.29%)	21 (42.85%)	0.109
	No	12 (38.70%)	28 (57.14%)	
Stage of the arterial hypertension	I	4 (13.55%)	1 (2.04%)	0.079
	II	6 (19.35%)	14 (28.57%)	
	III	9 (29.03%)	7 (14.28%)	
Degree of hypertension	Degree 1	10 (32.25%)	9 (18.36%)	0.098
	Degree 2	3 (9.67%)	10 (20.40%)	
	Degree 3	6 (19.35%)	3 (6.12%)	
Risk of cardiovascular disease	Low	10 (32.25%)	19 (38.77%)	0.449
	Moderate	8 (25.80%)	16 (34.70%)	
	High	8 (25.80%)	10 (20.40%)	
	Very high	5 (16.12%)	3 (6.12%)	
SCAD from anamnesis	Yes	4 (12.90%)	1 (2.04%)	<0.001 *
	No	2 (6.45161%)	25 (51.02041%)	
	Missing data	25 (80.64%)	23 (46.93%)	
Blood pressure reaction type on stress test	Asthenic	2 (10.5%)	2 (4.08%)	0.072
	Hypotonic	3 (15.8%)	1 (2.04%)	
	Hypertonic	1 (5.3%)	4 (8.16%)	
	Mild Hypertonic	1 (5.3%)	0 (0.0%)	
	Normotonic	12 (63.2%)	42 (85.71%)	
Functional class by Watt	FC-I	1 (3.22%)	7 (14.28%)	0.313
	FC-II	3 (9.67%)	6 (12.24%)	
	No SCAD	27 (87.09%)	36 (73.46%)	
Functional class by METs	FC-I	1 (3.22%)	5 (10.20%)	0.556
	FC-II	3 (9.67%)	7 (14.28%)	
	FC-III	0 (0.0%)	1 (2.04%)	
	No SCAD	27 (87.09%)	36 (73.46%)	
Type of reaction to the stress test (positive/negative)	Negative	16 (51.61%)	26 (53.06%)	0.191
	Suspected	11 (35.45%)	10 (20.40%)	
	Positive	4 (12.90%)	13 (26.53%)	
Reason of discontinuation of the stress test	Horizontal ST depression > 1 mm	2 (6.45%)	6 (12.24%)	0.385
	Reach goal HR	29 (93.54%)	42 (88.11%)	
Tolerance to exertion on stress test	Low	1 (3.22%)	1 (2.04%)	0.416
	Moderate	20 (64.45%)	23 (46.93%)	
	Close to high	3 (9.67%)	5 (10.20%)	
	High	3 (9.67%)	13 (26.53%)	
	Very high	4 (12.90%)	7 (14.28%)	
CAVI degree	Normal (<8)	9 (29.03%)	27 (55.10%)	0.073
	Borderline (8–9)	11 (35.48%)	11 (22.44%)	
	Pathological (>9)	11 (35.48%)	11 (22.44%)	
ABI degree	Normal	29 (93.54%)	47 (95.91%)	0.893
	Borderline	1 (3.22%)	1 (2.04%)	
	Abnormal	1 (3.22%)	0 (0.00%)	
	Non-compressible	0 (0.00%)	1 (2.04%)	
Biological estimated vascular age	Normal	16 (51.61%)	29 (59.18%)	0.507
	High	15 (48.38%)	20 (40.81%)	
CKD stage	I	12 (38.70%)	23 (46.93%)	0.285
	II	16 (51.61%)	25 (51.02%)	
	IIIa	3 (9.67%)	1 (2.04%)	
(**B**)				
Variable	Group 1 (*n* = 31). Positive stress-induced myocardial perfusion defect on CTP. Mean ± Std. dev.	Group 2 (*n* = 49). Negative stress-induced myocardial perfusion defect on CTP. Mean ± Std. dev.	t-value	*p*
Age	59.9307 ± 11.70846	53.9675 ± 9.23135	2.53382	0.013287 *
Pulse rest	70.2258 ± 10.74464	70.3265 ± 8.84446	−0.04562	0.963726
SBP rest	124.4839 ± 20.56351	122.3265 ± 11.22755	0.60653	0.545921
DBP rest	82.3548 ± 13.16447	79.5102 ± 9.81521	1.10453	0.272758
Body weight	77.1290 ± 14.71675	78.4204 ± 17.25762	−0.34465	0.731287
Height	169.8065 ± 9.41424	170.0408 ± 8.54695	−0.11487	0.908845
BMI	26.7076 ± 4.23191	27.0654 ± 5.31845	−0.31627	0.752643
Pulse rest	69.6452 ± 11.51390	69.6939 ± 7.63273	−0.02278	0.981884
Pulse after stress	85.7419 ± 11.88828	87.6122 ± 9.72072	−0.76835	0.444602
Goal heart rate	160.0693 ± 11.70846	166.0325 ± 9.23135	−2.53382	0.013287 *
Max HR	142.6774 ± 17.66614	148.5102 ± 10.91849	−1.82761	0.071432
Reached %	89.4238 ± 12.09432	89.6020 ± 6.84428	−0.08417	0.933138
WT	120.9677 ± 47.03579	128.5714 ± 42.38956	−0.74903	0.456093
METs	6.3290 ± 2.03571	6.8878 ± 1.92934	−1.23526	0.220441
EF (%)	64.4783 ± 4.77560	64.5476 ± 4.22075	−0.06046	0.951981
eVessel age	61.2258 ± 12.42232	53.5102 ± 13.44762	2.57376	0.011955 *
R-CAVI	8.5806 ± 1.03904	7.9714 ± 1.51891	1.95970	0.053603
L-CAVI	8.4387 ± 0.93868	8.0184 ± 1.46922	1.41852	0.160024
Mean CAVI	8.509677 ± 0.97506	7.994898 ± 1.48991	1.704581	0.092253
RABI	1.1232 ± 0.10041	1.1629 ± 0.07697	−1.99089	0.049996 *
LABI	1.1297 ± 0.08716	1.1584 ± 0.08032	−1.50589	0.136133
Mean SBP B	137.9839 ± 15.43399	132.0918 ± 10.91953	1.99878	0.049116
Mean DBP B	86.9194 ± 8.76841	84.2347 ± 8.00397	1.40836	0.162997
BP RB (=(SBP + DBP)/2)	106.4194 ± 12.85243	102.4286 ± 10.48411	1.51834	0.132972
BP LB (=(SBP + DBP)/2)	107.0968 ± 11.51044	102.9184 ± 9.63898	1.75092	0.083892
Mean BP B	106.7581 ± 11.94004	102.6735 ± 9.75990	1.67101	0.098728
BP RA (=(SBP + DBP)/2)	110.6452 ± 13.09338	106.9592 ± 12.11741	1.28474	0.202687
BP LA (=(SBP + DBP)/2)	111.2903 ± 12.77026	106.9388 ± 10.43913	1.66443	0.100039
Mean BP A	110.9677 ± 12.37129	106.9490 ± 10.69572	1.54024	0.127550
Mean ABI	1.1265 ± 0.09089	1.1606 ± 0.07259	−1.85781	0.066970
RTb	77.2581 ± 10.23708	82.5918 ± 14.85193	−1.75167	0.083761
LTb	74.1613 ± 12.88176	79.2245 ± 14.46730	−1.58966	0.115958
Mean Tb	75.7097 ± 10.65033	80.9082 ± 13.32318	−1.83215	0.070746
Right Tba	81.1935 ± 16.27968	89.4694 ± 15.52029	−2.27998	0.025341 *
Left Tba	83.0000 ± 14.73092	88.5510 ± 14.39048	−1.66560	0.099806
Mean Tba	82.0968 ± 15.38420	89.0102 ± 14.87850	−1.99834	0.049164 *
Lha (cm)	148.0571 ± 7.65284	148.2476 ± 6.94782	−0.11487	0.908845
haPWV (m/s)	0.9533 ± 0.11852	0.8860 ± 0.12080	2.44529	0.016727 *
β-stiffness index from PWV	2.9538 ± 0.65907	2.7567 ± 0.89559	1.05682	0.293858
Creatinine (µmol/L)	80.3787 ± 15.44759	84.2339 ± 16.34146	−1.04969	0.297103
eGFR (2021 CKD-EPI Creatinine)	84.8548 ± 15.14628	85.5898 ± 14.53585	−0.21677	0.828950

**Table 5:** The quality of the bicycle ergometry appeared quite low in our cohort.

Chars	Point Estimate	95% CI
AUC	0.507	[0.388; 0.625]
Sensitivity	0.484	[0.306; 0.657]
Specificity	0.531	[0.392; 0.673]
Negative predictive value	0.619	[0.465; 0.758]
Positive predictive value	0.395	[0.238; 0.553]

**Table 6:** The 10 most statically significant features according to the build model are represented in the table.

№	Feature	Lasso Absolute Coefficient
1.	dltlos_13_94_0537307640158	0.0153039
2.	dltlos_13_144_91780932607332	0.00704803
3.	dltlos_13_87_93367781464133	0.00416145
4.	dltlos_13_87_07684847044642	0.00393754
5.	dltlos_03_72_05367879285716	0.00388224
6.	dltlos_01_100_0459797175948	0
7.	dltlos_13_447_1045512664764	0
8.	dltlos_13_374_08597586892273	0
9.	dltlos_13_375_07957930255367	0
10.	dltlos_13_429_0881741701056	0

**Table 7:** The quality of the exhaled breath biomarkers in the diagnosis of ischemic heart disease.

Chars	Point Estimate	95% CI
AUC	0.838	[0.73655857; 0.91493173]
Sensitivity	0.839	[0.692; 0.964]
Specificity	0.776	[0.666; 0.889]
Negative predictive value	0.884	[0.771; 0.975]
Positive predictive value	0.703	[0.559; 0.849]

**Table 8:** Comparative presentation of the most significant VOCs in the exhaled breath analysis of individuals with ischemic heart disease and without ischemic heart disease. The presented *m*/*z* (mass/charge) ratios in our study are close to *m*/*z* ratios published in the literature or from the chemical substances’ library provided by the manufacturer of the IONICON PTR-TOF-MS device. In case that the *m*/*z* ratio does not have a known chemical name, represented as a chemical formula. After filtering the list of the *m*/*z* ratio from the artifacts and duplicates values, out of 10 top *m*/*z* ratios remained 5 *m*/*z* ratios. * Can be two chemical substances according to the found *m*/*z*.

*m*/*z* in Our Study	Name of the Chemical Substance	Annotation Method	Error, ppm	Coefficient	References
72.0537	C_4_H_8_O^+^	Ionicon Database	−46	0.00388224	
87.0768	2-Pentanone or 3-methyl-2-butanone *	N/A	−41.8	0.00393754	Kos R. et al. (2022) [62]
87.9337	N/A	N/A		0.00416145	
94.0537	C_2_H_7_NO_3_H^+^	Ionicon Database	39.6	0.01530394	
144.9178	N/A	N/A		0.00704803	
Water PA = 691 kJ/mol					

## Data Availability

Applicable on reasonable request.

## Supplementary Materials

The following supporting information can be downloaded at: https://www.mdpi.com/article/10.3390/biomedicines12122814/s1, Supplementary S1: The comparative features of the sample divided by various binary categorical variables. The represented continuous variables are all statistically significant at *p* < 0.05.

## Abbreviations

CVD: cardiovascular disease; CTP: stress computed tomography myocardial perfusion imaging; VOCs: volatile organic compounds.
