# Supplementary material for: Machine Learning Model Discriminate Ischemic Heart Disease Using Breathome Analysis
Source: Biomedicines. 2024 Dec 11;12(12):2814. doi: 10.3390/biomedicines12122814 (PMC11673773; doi:10.3390/biomedicines12122814)
Supplement: Supplementary file 1 [file biomedicines-12-02814-s001.zip › biomedicines-3320187-supplementary.pdf]

Supplementary S1: The comparative features of the sample divided by various binary categorical variables. The represented continuous variables are all statistically significant at $p<0.05$.

A

Variable	T-tests; Grouping: Gender (Both in primary data.stw)<br>Group 1: F<br>Group 2: M			
	Mean F	Mean M	t-value	p
Body weight	71.5974	83.9341	-3.65280	0.000468
Height	163.9359	175.6707	-7.93010	0.000000
WT	94.8718	154.8780	-8.28672	0.000000
METs	5.8846	7.4195	-3.74534	0.000343
Lha (cm)	143.2849	152.8242	-7.93010	0.000000
β-stiffness index from PWV	2.6430	3.0138	-2.08085	0.040730
Creatinine (μmol/L)	72.4862	92.4937	-7.13522	0.000000

B

Variable	T-tests; Grouping: Smoking (Both in primary data.stw)<br>Group 1: No<br>Group 2: Yes			
	Mean No	Mean Yes	t-value	p
DBP rest	79.4394	86.1429	-2.06890	0.041869
Body weight	75.3121	90.2143	-3.31009	0.001414
Height	168.9848	174.5000	-2.17081	0.032987
BMI	26.3829	29.4905	-2.20717	0.030242
Lha (cm)	147.3892	151.8725	-2.17081	0.032987

C

Variable	T-tests; Grouping: Concomitant disease (Both in primary data.stw)<br>Group 1: Yes<br>Group 2: No			
	Mean Yes	Mean No	t-value	p
Age	58.2005	53.0605	2.15911	0.034084
BMI	28.2638	25.4612	2.54843	0.012894
Goal heart rate	161.7995	166.9395	-2.15911	0.034084
WT	115.8537	139.2857	-2.35097	0.021390
METs	6.0683	7.5000	-3.29168	0.001528
eVessel age	59.4146	52.0286	2.42323	0.017829
Mean SBP B	136.6829	130.7429	2.02434	0.046545
BP RA (=(SBP+DBP)/2)	110.7561	104.0571	2.42353	0.017815
BP LA (=(SBP+DBP)/2)	111.2195	103.9143	3.02442	0.003423

Mean BP A	110.9878	103.9857	2.85424	0.005593
RTb	77.7805	84.6286	-2.24257	0.027920
Mean Tb	76.3780	82.7714	-2.24909	0.027482

D

Variable	T-tests; Grouping: Atherosclerosis of the coronary artery (Both in primary data.stw)<br>Group 1: No<br>Group 2: Yes			
	Mean No	Mean Yes	t-value	p
Age	51.2944	64.1558	-6.53443	0.000000
Body weight	74.1694	83.8484	-2.69905	0.008522
Goal heart rate	168.7056	155.8442	6.53443	0.000000
Reached %	87.7888	92.2900	-2.19067	0.031461
eVessel age	50.1224	66.5806	-6.56896	0.000000
R-CAVI	7.6796	9.0419	-4.89037	0.000005
L-CAVI	7.7122	8.9226	-4.53296	0.000021
Mean SBP B	131.3673	139.1290	-2.68406	0.008879
BP RB (=(SBP+DBP)/2)	101.1020	108.5161	-2.92887	0.004458
BP LB (=(SBP+DBP)/2)	102.0000	108.5484	-2.82609	0.005982
Mean BP B	101.5510	108.5323	-2.95961	0.004078
LTb	79.7143	73.3871	2.00482	0.048452
Right Tba	91.5714	77.8710	4.01447	0.000136
Left Tba	91.1224	78.9355	3.93370	0.000180
Mean Tba	91.3469	78.4032	4.00722	0.000139
haPWV (m/s)	0.8614	0.9922	-5.36049	0.000001
β-stiffness index from PWV	2.5866	3.2226	-3.66533	0.000449
Creatinine (μmol/L)	79.2967	88.1826	-2.49669	0.014644
eGFR (2021 CKD-EPI Creatinine)	88.9265	79.5806	2.90054	0.004838

E

Variable	T-tests; Grouping: Hemodynamically significant (>60%) (Both in primary data.stw)<br>Group 1: No<br>Group 2: Yes			
	Mean No	Mean Yes	t-value	p
Age	54.6859	68.8402	-4.14128	0.000087
Goal heart rate	165.3141	151.1598	4.14128	0.000087
Max HR	148.2254	130.6667	3.80483	0.000281
EF (%)	65.1207	59.5714	3.41368	0.001125
eVessel age	54.4085	73.0000	-4.29401	0.000050
R-CAVI	7.9563	10.1889	-5.30446	0.000001

L-CAVI	7.9634	9.9000	-4.75273	0.000009
Mean SBP B	133.0423	144.8889	-2.65461	0.009621
Mean DBP B	84.4014	92.1667	-2.73106	0.007803
BP RB (=(SBP+DBP)/2)	102.6620	114.3333	-2.99746	0.003650
BP LB (=(SBP+DBP)/2)	103.5070	112.6667	-2.54098	0.013038
Mean BP B	103.0845	113.5000	-2.85369	0.005532
LTb	78.3803	68.4444	2.04390	0.044339
Mean Tb	79.8944	71.0000	2.04336	0.044394
Right Tba	88.9718	64.8889	4.72565	0.000010
Left Tba	88.7606	67.7778	4.50455	0.000023
Mean Tba	88.8662	66.3333	4.65823	0.000013
haPWV (m/s)	0.8871	1.1089	-6.13269	0.000000
β-stiffness index from PWV	2.6918	3.9474	-4.97558	0.000004

F

Variable	T-tests; Grouping: Myocardial perfusion defect before Stress ATP (Both in primary data.stw) Group 1: Yes Group 2: No				
	Mean Yes	Mean No	t-value	df	p
Max HR	138.4615	150.0000	-3.68868	78	0.000415
Reached %	86.4746	91.0055	-2.11588	78	0.037545
EF (%)	62.7000	65.3333	-2.30735	63	0.024333
eVessel age	61.8846	53.9074	2.55701	78	0.012498
LABI	1.1204	1.1602	-2.03157	78	0.045603
Right Tba	80.3846	89.0926	-2.30823	78	0.023637
Mean Tba	81.2115	88.7963	-2.11386	78	0.037723

G

Variable	T-tests; Grouping: Atherosclerosis in other arteries (Yes/No) (Both in primary data.stw) Group 1: No Group 2: Yes			
	Mean No	Mean Yes	t-value	p
Age	48.4591	61.8114	-6.67105	0.000000
SBP rest	116.8750	126.9024	-2.81141	0.006372
DBP rest	77.0313	82.8780	-2.21050	0.030294
Body weight	71.1656	80.9585	-2.71672	0.008276
BMI	24.4660	28.4944	-3.74890	0.000359

Goal heart rate	171.5409	158.1886	6.67105	0.000000
Max HR	152.4063	141.3415	3.45324	0.000938
WT	139.0625	114.6341	2.35423	0.021332
METs	7.9406	5.8195	5.19338	0.000002
eVessel age	46.3125	64.0000	-7.21404	0.000000
R-CAVI	7.5125	8.6780	-3.85771	0.000249
L-CAVI	7.5781	8.5707	-3.44625	0.000959
Mean SBP B	127.1406	139.8171	-4.41075	0.000036
Mean DBP B	80.3438	88.1463	-4.39502	0.000038
BP RB (=(SBP+DBP)/2)	97.2500	108.5122	-4.51995	0.000024
BP LB (=(SBP+DBP)/2)	99.3125	107.9512	-3.63760	0.000518
Mean BP B	98.2813	108.2317	-4.21410	0.000073
BP RA (=(SBP+DBP)/2)	101.0000	112.6098	-4.38235	0.000040
BP LA (=(SBP+DBP)/2)	101.7188	113.0000	-4.69401	0.000013
Mean BP A	101.3594	112.8049	-4.81404	0.000008
RTb	87.6563	76.0488	3.97243	0.000169
LTb	82.4063	74.5366	2.40142	0.018952
Mean Tb	85.0312	75.2927	3.48807	0.000839
Right Tba	94.5938	79.2439	4.35025	0.000045
Left Tba	93.8750	80.2927	4.22749	0.000069
Mean Tba	94.2344	79.7683	4.32624	0.000049
haPWV (m/s)	0.8353	0.9627	-4.96219	0.000005
β-stiffness index from PWV	2.4801	3.0110	-2.92002	0.004688

H

Variable	T-tests; Grouping: Carotid (Both in primary data.stw)<br>Group 1: No<br>Group 2: Yes			
	Mean No	Mean Yes	t-value	p
Age	49.3632	62.3779	-6.47581	0.000000
SBP rest	117.9412	127.0000	-2.50663	0.014480
DBP rest	76.9118	83.0256	-2.31854	0.023304
Body weight	72.7735	81.1103	-2.26866	0.026331
BMI	24.7749	28.6399	-3.55828	0.000670
Goal heart rate	170.6368	157.6221	6.47581	0.000000
Max HR	152.2353	141.2308	3.42535	0.001024
WT	137.5000	114.1026	2.24521	0.027870
METs	7.7676	5.7692	4.71014	0.000012
eVessel age	47.5294	64.6154	-6.85002	0.000000
R-CAVI	7.6265	8.7000	-3.49087	0.000832

L-CAVI	7.6912	8.5872	-3.06143	0.003109
Mean SBP B	127.3676	140.3974	-4.60363	0.000018
Mean DBP B	80.7059	88.2308	-4.22680	0.000069
BP RB (=(SBP+DBP)/2)	97.9118	108.8718	-4.34732	0.000045
BP LB (=(SBP+DBP)/2)	99.8529	108.2564	-3.51634	0.000767
Mean BP B	98.8824	108.5641	-4.05994	0.000125
BP RA (=(SBP+DBP)/2)	101.9412	112.8462	-4.04464	0.000132
BP LA (=(SBP+DBP)/2)	103.0000	113.2051	-4.02214	0.000142
Mean BP A	102.4706	113.0256	-4.27458	0.000059
RTb	86.6176	76.0256	3.55900	0.000669
LTb	81.5588	73.6154	2.57072	0.012246
Mean Tb	84.0882	74.8205	3.35620	0.001272
Right Tba	94.2059	78.6667	4.43953	0.000032
Left Tba	93.4118	79.8718	4.22990	0.000069
Mean Tba	93.8088	79.2692	4.37414	0.000041
haPWV (m/s)	0.8461	0.9674	-4.68414	0.000013
β-stiffness index from PWV	2.5549	3.0133	-2.48557	0.015291

I

Variable	T-tests; Grouping: Brachiocephalic (Both in primary data.stw)<br>Group 1: No<br>Group 2: Yes			
	Mean No	Mean Yes	t-value	p
Age	50.1003	62.3642	-5.92974	0.000000
SBP rest	118.3333	127.1081	-2.42735	0.017748
DBP rest	76.6667	83.5946	-2.66257	0.009586
Body weight	73.1472	81.1973	-2.19058	0.031767
BMI	24.9034	28.7238	-3.51942	0.000759
Pulse rest	66.5556	71.4865	-2.38704	0.019651
Goal heart rate	169.8997	157.6358	5.92974	0.000000
Max HR	151.4722	141.3784	3.10945	0.002697
WT	136.1111	114.1892	2.09958	0.039321
METs	7.6444	5.7811	4.31716	0.000050
Mean SBP B	128.6111	139.8919	-3.85507	0.000252
Mean DBP B	81.3889	87.9730	-3.60347	0.000579
BP RB (=(SBP+DBP)/2)	98.9444	108.4595	-3.66602	0.000472
BP LB (=(SBP+DBP)/2)	100.5000	108.0811	-3.13005	0.002537
Mean BP B	99.7222	108.2703	-3.50544	0.000794
BP RA (=(SBP+DBP)/2)	102.9167	112.4865	-3.46814	0.000894
BP LA (=(SBP+DBP)/2)	103.5278	113.2432	-3.79923	0.000303
Mean BP A	103.2222	112.8649	-3.83495	0.000269
RTb	85.6111	76.4324	3.02549	0.003455
LTb	80.6111	74.1081	2.07795	0.041331
Mean Tb	83.1111	75.2703	2.78450	0.006867
Right Tba	92.5000	79.4865	3.58232	0.000620
Left Tba	92.3333	80.1892	3.71368	0.000403

Mean Tba	92.4167	79.8378	3.67203	0.000463
haPWV (m/s)	0.8603	0.9601	-3.68383	0.000445
β-stiffness index from PWV	2.6092	2.9852	-2.01532	0.047658

J

Variable	T-tests; Grouping: Arterial Hypertension (Both in primary data.stw)<br>Group 1: No<br>Group 2: Yes			
	Mean<br>No	Mean<br>Yes	t-value	p
Age	51.4730	61.0834	-4.52681	0.000021
SBP rest	117.8250	128.5000	-3.27783	0.001564
DBP rest	77.7750	83.4500	-2.32017	0.022947
BMI	25.6294	28.2240	-2.44082	0.016921
Goal heart rate	168.5270	158.9166	4.52681	0.000021
WT	137.5000	113.7500	2.48550	0.015076
METs	7.4750	5.8675	3.95860	0.000165
eVessel age	49.3250	63.6750	-5.57881	0.000000
R-CAVI	7.6525	8.7625	-3.91296	0.000194
L-CAVI	7.7225	8.6400	-3.35647	0.001222
Mean SBP B	128.4625	140.2875	-4.50838	0.000023
Mean DBP B	82.8125	87.7375	-2.74183	0.007574
BP RB (=(SBP+DBP)/2)	99.0250	108.9250	-4.22291	0.000065
BP LB (=(SBP+DBP)/2)	101.3500	107.7250	-2.82342	0.006028
Mean BP B	100.1875	108.3250	-3.62953	0.000505
BP RA (=(SBP+DBP)/2)	104.4750	112.3000	-2.91720	0.004611
BP LA (=(SBP+DBP)/2)	103.7500	113.5000	-4.15705	0.000082
Mean BP A	104.1125	112.9000	-3.69048	0.000413
RTb	84.8250	76.2250	3.00316	0.003590
LTb	80.7750	73.7500	2.30228	0.023987
Mean Tb	82.8000	74.9875	2.91367	0.004659
Right Tba	93.9250	78.6000	4.76792	0.000008
Left Tba	92.6750	80.1250	4.20660	0.000069
Mean Tba	93.3000	79.3625	4.53289	0.000021
haPWV (m/s)	0.8550	0.9692	-4.63729	0.000014
β-stiffness index from PWV	2.6538	3.0123	-2.00831	0.048072

K

Variable	T-tests; Grouping: SCAD II-III (Both in primary data.stw)<br>Group 1: No<br>Group 2: Yes			
	Mean<br>No	Mean<br>Yes	t-value	P
Age	51.3686	74.1726	-4.50496	0.000094

Height	172.5517	161.6667	2.14298	0.040345
Pulse after stress	86.3448	70.0000	3.15103	0.003673
Goal heart rate	168.6314	145.8274	4.50496	0.000094
Max HR	150.9655	170.3333	-2.18173	0.037097
Reached %	89.6496	116.6267	-4.95212	0.000027
eVessel age	50.1034	74.6667	-3.35831	0.002146
R-CAVI	7.8897	11.2667	-4.10892	0.000283
L-CAVI	7.9207	11.1333	-4.10755	0.000284
Right Tba	94.0345	49.3333	5.57398	0.000005
Left Tba	93.3103	51.6667	5.70435	0.000003
Mean Tba	93.6724	50.5000	5.66552	0.000004
Lha (cm)	150.2887	141.4403	2.14298	0.040345
haPWV (m/s)	0.8723	1.1609	-4.12256	0.000272
β-stiffness index from PWV	2.7431	4.4903	-3.29422	0.002537
eGFR (2021 CKD-EPI Creatinine)	88.0345	67.4000	2.32528	0.027008

L

Variable	T-tests; Grouping: Reason of discontinuation (Both in primary data.stw) Group 1: Reach goal HR Group 2: Horizontal ST depression >1mm			
	Mean Reach goal HR	Mean Horizontal ST depression >1mm	t-value	P
Pulse after stress	87.9014	77.4286	2.55692	0.012554
R-CAVI	8.0873	9.8286	-3.40533	0.001058
L-CAVI	8.0873	9.5571	-3.01437	0.003499
Right Tba	86.9859	73.1429	2.23774	0.028167
Mean Tba	86.9155	74.4286	2.13132	0.036296
haPWV (m/s)	0.9027	1.0327	-2.75026	0.007439
β-stiffness index from PWV	2.7566	3.7569	-3.27037	0.001616

M

Variable	T-tests; Grouping: vessel stiffness (Both in primary data.stw) Group 1: Normal Group 2: High			
	Mean Normal	Mean High	t-value	p
Age	54.0870	59.0955	-2.14345	0.035193
SBP rest	119.4667	127.9143	-2.50815	0.014212
Goal heart rate	165.9130	160.9045	2.14345	0.035193

Max HR	149.8667	141.6000	2.70090	0.008479
WT	135.0000	113.5714	2.20767	0.030206
METs	7.1889	6.0057	2.76413	0.007119
eVessel age	50.0667	64.7714	-5.71036	0.000000
R-CAVI	7.6156	8.9686	-4.96292	0.000004
L-CAVI	7.6311	8.8886	-4.87258	0.000006
BP RA (=(SBP+DBP)/2)	105.6000	111.9714	-2.31324	0.023345
BP LA (=(SBP+DBP)/2)	106.0667	111.9143	-2.31365	0.023322
Mean BP A	105.8333	111.9429	-2.43662	0.017104
RTb	85.0889	74.6571	3.71174	0.000384
Mean Tb	82.2222	74.6143	2.80499	0.006349
Right Tba	90.7556	80.4857	2.94006	0.004316
Left Tba	90.5333	81.0857	2.99525	0.003674
Mean Tba	90.6444	80.7857	2.98776	0.003756
haPWV (m/s)	0.8652	0.9723	-4.23338	0.000062
β-stiffness index from PWV	2.5951	3.1390	-3.12731	0.002480
